# Screening of Suitable Ionic Liquids as Green Solvents for Extraction of Eicosapentaenoic Acid (EPA) from Microalgae Biomass Using COSMO-RS Model

**DOI:** 10.3390/molecules24040713

**Published:** 2019-02-16

**Authors:** Shiva Rezaei Motlagh, Razif Harun, Dayang Radiah Awang Biak, Siti Aslina Hussain, Wan Azlina Wan Ab Karim Ghani, Ramin Khezri, Cecilia Devi Wilfred, Amal A. M. Elgharbawy

**Affiliations:** 1 Department of Chemical and Environmental Engineering, Faculty of Engineering, University Putra Malaysia, UPM, Serdang, Selangor 43400, Malaysia; shiva.rezaei.m@gmail.com (S.R.M.); dradiah@upm.edu.my (D.R.A.B.); aslina@upm.edu.my (S.A.H.); wanazlina@upm.edu.my (W.A.W.A.K.G.); ramin.khezri@gmail.com (R.K.); 2 Department of Fundamental and Applied Sciences, Centre of Research in Ionic Liquids (CORIL), Universiti Teknologi Petronas, UTP, Bandar Seri Iskandar, Perak 32610, Malaysia; cecili@utp.edu.my; 3 International Institute for Halal Research and Training (INHART), International Islamic University Malaysia, Gombak, Kuala Lumpur 50728, Malaysia; amalgh@iium.edu.my

**Keywords:** COSMO-RS, omega-3, EPA extraction, ionic liquids, screening, extraction capacity, infinite dilution activity coefficient

## Abstract

Omega-3 poly unsaturated fatty acids (PUFA) particularly eicosapentaenoic acid (EPA), and docosahexaenoic acid (DHA), have many health benefits including reducing the risk of cancer and cardiovascular disease. Recently, the use of ionic liquids (ILs) in lipid extraction from microalgae provides the potential to overcome common drawbacks, offers several other benefits. To date, very limited researches are available to focus on extracting microalgae lipid and PUFA in particular by using ILs. The objective of current work is to screen the potential ILs that can be applied in EPA extraction. In this study, fast ILs screening was performed with the help of a conductor like screening model for real solvents (COSMO-RS) and the ILs with higher capacity values for use in extraction of EPA were compared. According to the results, the highest capacity for EPA extraction among 352 screened cation/anion combinations belongs to [TMAm][SO_4_]. It is expected to achieve a higher yield of EPA once applying this combination as the solvent in the process of extraction. ILs with small anions were observed to have higher capacities, as well possessing higher charge density compared to larger ones, and therefore, they are more preferable for extraction purposes. Moreover, shorter alkyl chain cations are preferred when using imidazolium-based IL, which agrees with experimental data.

## 1. Introduction

Health benefits provided by omega-3 polyunsaturated fatty acids (PUFAs), including eicosapentaenoic acid (EPA) and docosahexaenoic acid (DHA) in particular are noteworthy to the society as it reduces the risk of cancer, autoimmune, cardiovascular disease, inflammatory disorders, cystic fibrosis, disrupted neurological function, bowel disease, and mental illness [1,2,3,4,5,6]. The use of PUFA as health prospects has gained great attention in last years. Current dietary health organization recommends a daily intake of 250–500 mg of EPA + DHA for primary and secondary prevention of coronary heart disease [7,8]. Additionally, according to Shahidi and Ambigaipalan human bodies requires the supplementary sources of EPA and DHA as they are vulnerable to shortages of necessary enzymes to place a double bond at the omega-3 position and hence are unable to synthesize them to a sufficient extent [9]. Those are one of the two classes of essential fatty acids that have such characteristics and therefore must be taken from diet and supplements. Thus, the external source of omega-3 PUFA is necessary. 

Lately, the main supply of both EPA and DHA are provided from fish, however, the intake of fish oil for long period may cause a deficiency of vitamin E due to the high level of vitamin A and D involved in fish lipids [10,11]. Many harmful contaminants such as methylmercury, copper, and organic pollutants as polychlorinated biphenyls (PCBs) or dioxins are also found in some species of fish especially in salmon, sardine, anchovy, and tuna, which may impose toxic effects on human health [11,12], and therefore, alternative sources of omega-3 PUFA are required. Microalgae oil and fish oil have the same amount of omega-3 but microalgae oil has more advantages in terms of health and less toxicity compared to fish oil [13]. The oil derived from microalgae contains squalene and phytosterols, which indicates several health benefits to the human body and it not having an unpleasant smell and cholesterol. The process of converting microalgae into omega-3 PUFA mainly consists of four steps, including microalgae cultivation, harvesting, cell disruption (lipid extraction), and transesterification (GC/FID analysis). There are numerous methods that have been used for lipid extraction, but most of them using conventional solvents, which are mostly volatile, aromatic, thermally unstable, and environmentally unfriendly, in addition to causing health and safety problems [14]. For instance, the use of solvents such as hexane or isopropanol in Soxhlet extraction likewise chloroform, methanol, and water in the Bligh and Dyer method (both methods are considered to be the most widely used techniques for lipid extraction) introduced a few concerns due to the toxicity associated with the solvents that brings biosafety issues and is required to be replaced by environmentally friendly, biocompatible, and less toxic solvents.

Therefore, solvent extraction using ionic liquids (ILs) are preferable and has potential in the extraction of the microalgae lipids as an alternative to traditional solvents that use toxic solvent, long duration time, and demand a huge amount of energy [15]. In solvent extraction, it is crucial to have high capacity to extract the targeted solute. ILs are salts with low melting point and consist of large asymmetric organic cations and smaller organic or inorganic anions [16]. The cations of ILs are generally composed of nitrogen contacting ring like imidazolium, pyrrolidinium to which different functional groups can be added [17]. Additionally, ILs are often considered to be “green” solvents because of their very low vapor pressure, great thermal stability with a broad electrochemical window, and high ionic conductivity compared to conventional solvents [18]. In a study by Pan et al. [19], the microwave-assisted extraction with [BMIM][HSO_4_] as IL was observed to have higher yield rather than the conventional method of oil bath using an organic solvent for the extraction of lipid from *Nannochloropsis salina*, *Chlorella sorokiniana,* and *Galdieria sulphuraria*. Moreover, Kim et al. [20] used the mixture of IL like [BMIM][CF_3_SO_3_] with methanol to extract lipid (biodiesel purpose) from microalgae *C.vulgaris,* which obtained 19%. According to the literature, ILs in general have prominent advantages over organic solvent like hexane, chloroform, however, there are only a few studies available to use ILs for extraction of long chain fatty acids such as EPA and DHA. It is noticeable that using ILs for processing is costly and time-consuming, and thus a reasonable screening of different cations and anions combination is obligatory prior to selecting the appropriate ILs. Therefore, it seems crucial to predict ILs behavior before the experimental application. The conductor like screening model for real solvent (COSMO-RS) proposed by Klamt is a model that can be used for such fast prior prediction [21,22]. COSMO-RS is a new method to predict the thermodynamic properties of fluid and liquid mixture based on quantum chemistry concept of density functional theory (DFT) [23]. Chosen the suitable IL for the extraction efficiency, some thermodynamic properties are important. The solvent capacity, selectivity at infinite dilution and performance index was used many times by previous studies for solvent liquid-liquid extraction prediction, which was calculated under the activity coefficient at the infinite dilution. For instance, Mansoure et al. [24] used the COSMO-RS tool for the prediction of ammonium ILs capacity and then selectivity values for β-carotene separation from n-hexane. Additionally, the study reported by Zeeshan et al. [25] COSMO-RS software used for the calculation of activity coefficient at infinite dilution and then selectivity, capacity, and performance index to predict different types of ILs potential for asphaltene extraction as a part of liquid-liquid extraction. Moreover, in a recent study by Xing et al. [26], it has been used COSMO-RS for prediction and exploring the potential separation mechanism of long chain fatty acids (EPA, DHA, CLAs, OA, and SA) through the calculation of different solvents capacity, selectivity, and performance index values. In another study, adjustable parameters were re-optimized in COSMO-RS to fit for the systems containing ionic liquids (ILs), whereby a vast numbers of activity coefficients and at infinite dilution and CO_2_ solubility were collected from references and used as a training set. The results showed that the predicted results by COSMO-RS model with the new optimized parameters are in agreement with experimental data [27]. As pointed out by previous literatures, COSMO-RS only require the structural information of interacting species in order to calculate their related thermodynamic properties through a set of defined mathematical equations and hence one does not need to perform any experimentations in advance [28,29]. It is noteworthy to mention that, although COSMO-RS is a powerful screening tool, this software has been addressed by a number of authors as a mean field model that does not account for solvent molecular structure and correlations. Therefore, it is suggested that when using COSMO-RS, the important effects of explicit interactions between organic molecules and water, which can lead to charge transfer between the solute and water, must also be considered [30,31,32,33,34].

While the ILs screening potential seems crucial for extraction of omega-3 (EPA, DHA and ALA) compounds from biomass, however to the best of the author’s knowledge, no significant report has been published yet to concern the application of COSMO-RS in that particular regard. Therefore, this study strictly help and open a new horizon about ILs interaction toward valuable omega-3 compound for researchers who seek to replace the conventional solvent with proper green solvent (ILs) without any requirement to perform expensive and time consuming lab experimentations. Moreover, what makes the COSMO-RS screening even more valuable is that it provides the facility to study over the variety of 352 combination of cations and anions as ILs to act as solvents in interaction with EPA compounds.

In this study, COSMO-RS has been used as the screening methodology to select the most suitable ILs as a part of solid-liquid extraction of EPA from microalgae. This work aims to predict infinite capacity values of ILs for EPA extraction and without the use of any experimental data.

## 2. Results and Discussion

The principle of COSMO-RS as a physical model considers the interaction energy of polarization charge densities between the sigma bonds. Net surface charge of EPA is calculated in terms of Sigma profile (σ-profile), which is related to the charge density profiles of molecular surface and Sigma potential (σ-potential) in order to clear its chemical nature. The polarity of the molecule is typically determined by the help of molecular electrostatic potentials. The best approach to investigate the molecular similarity is through comparing the electron density or electrostatic potential that is created by molecules, considering all space within the van der Waals surfaces of the molecules or within all regions outside the molecular cores [35]. The interactions of the electrostatic (polarity) among IL systems will integrate the hydrogen-bonding and hydrophobicity of a structure in the method of COSMO-RS. Therefore, it demonstrates multiple solvation interactions of ILs and presents the quantity and clear visual of polarity distribution on molecular surfaces with the help of sigma (σ) profiles [36]. According to the σ-profile, higher absolute values of σ indicates the stronger compound as a hydrogen bond donor or a hydrogen bond acceptor [37].

**Figure 1:**
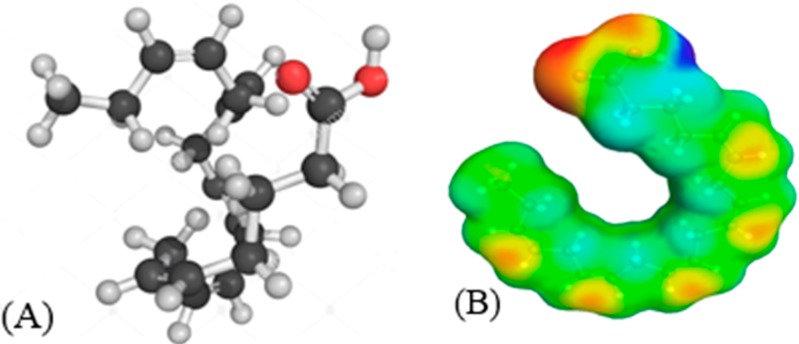
(**A**) EPA chemical structure and (**B**) sigma surface.

**Figure 2:**
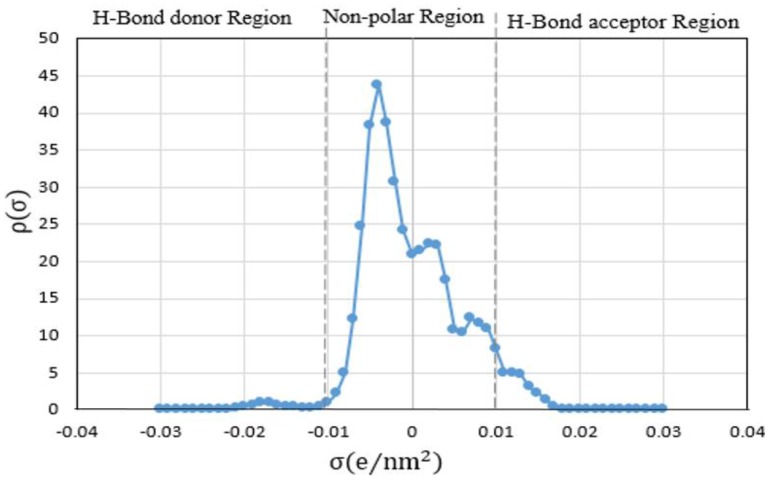
The σ-profile of EPA was obtained by COSMO-RS.

**Figure 3:**
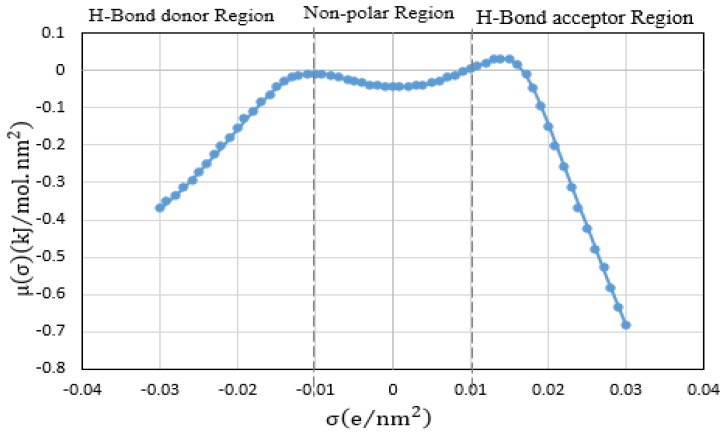
The σ-potential of EPA was obtained by COSMO-RS.

The chemical structure of EPA and its sigma surface are illustrated in Figure 1. The results of σ-profile and σ-potential are indicated in Figure 2 and Figure 3, respectively. Significant terms related to qualitative description of EPA molecules including hydrogen bonding, polarity, and lipophilicity/hydrophilicity can be visualized with the help of COSMO-RS 3D screening charge distribution (sigma surface). Charge distribution is coded in colors such as red color, which represents the negative (−ve) charge, whereas blue and green represents the positive (+ve) and neutral (0) charge on the molecular surface, respectively. The screening charge density surface (s-surface) contains all relevant information for COSMO-RS to calculate the chemical potential.

**Figure 4:**
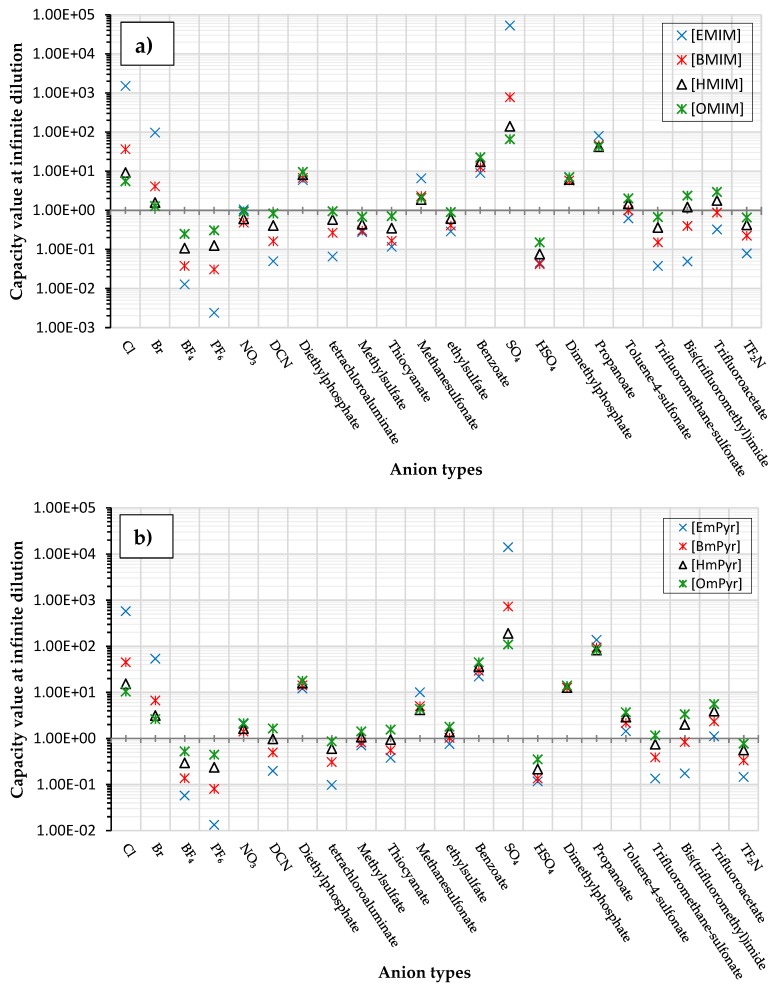

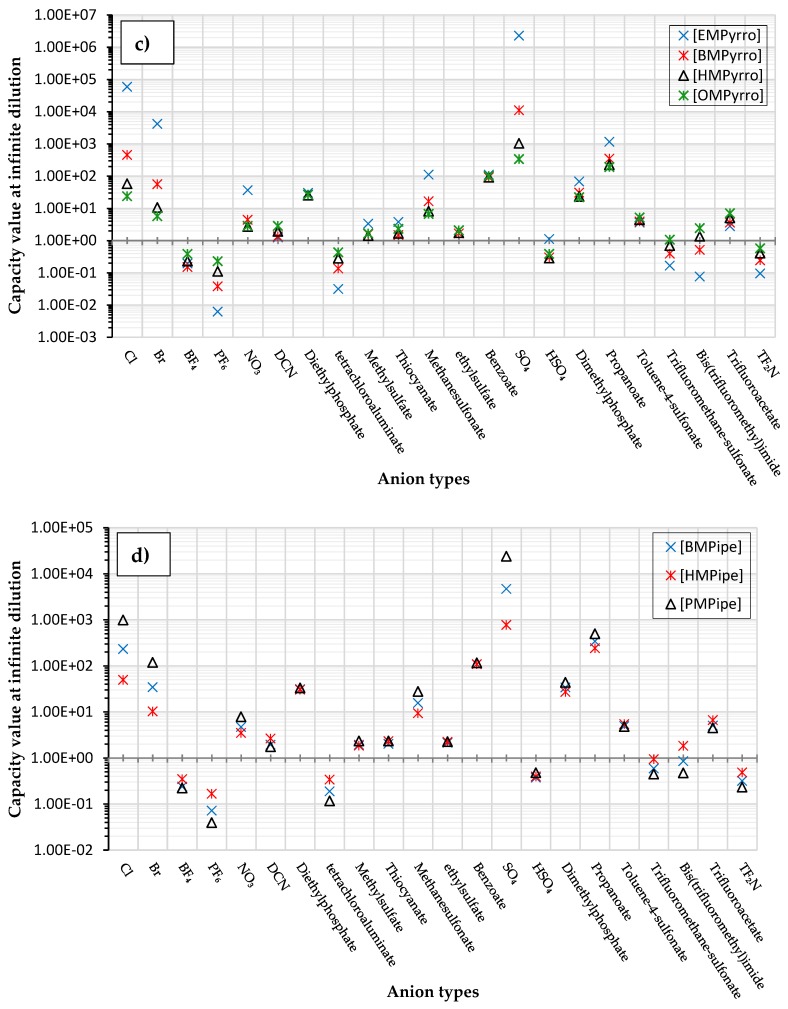

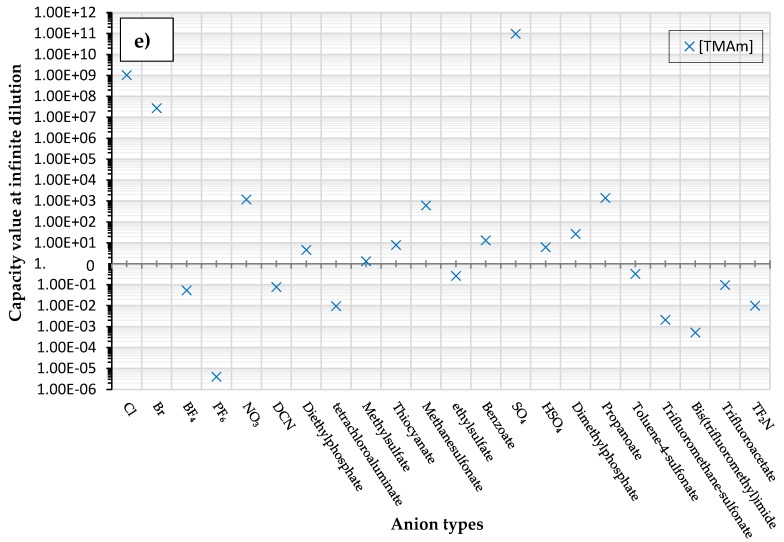
The capacity values at infinite dilution of (**a**) Imidazolium, (**b**) pyridinium, (**c**) Pyrrololidinium, (**d**) piperidinium, and (**e**) tetra-methyl ammonium cations alkyl chain length with 22 anion types for EPA extraction at T = 298.15 K.

The capacity values related to five types of cation-base ILs including imidazolium, pyridinium, pyrrolidinium, piperidinium, and tetra-methyl ammonium with 22 types of anions were screened at 298.15 K and the results are summarized in Figure 4a–e. The results are followed by recommendations for the selection of suitable ILs to be applied in EPA extraction.

Generally, sigma profile divided into three important parts. The first part, where σ < −0.01 e/nm^2^, shows the hydrogen bond donor part. The second part extending between −0.01 < σ < +0.01 e/nm^2^, which belongs to the non-polarized area and the third part, where σ > +0.01 e/nm^2^, shows the hydrogen bond acceptor part. It is remarkable that there are a series of sharp a peak in the non-polarized area and there is also a slight peak in hydrogen bond acceptor and donor. Consequently, EPA is highly non-polar and is due to all of the peaks merely crowded in the non-polar region. 

Chemical potential or sigma potential (μ(σ)) demonstrate the deep understanding about which ILs structures have a high impact on interaction with EPA molecules. Sigma potential (μ(σ)) like sigma profile is divided into three important areas. The first zone where σ < −0.01 e/nm^−2^ belongs to the interaction with hydrogen bond donors. The second zone where −0.01< σ < 0.01 e/nm^−2^ is related to non-polar area and the third zone, where σ > 0.01 e/nm^−2^ belongs to the hydrogen bond acceptor part. Sigma potential shows that EPA can interact with a polar compound due to the peaks located in negative values on the y axis from 0 < (μ(σ)) < −0.7. Additionally it has high attraction toward both hydrogen-bond donor and acceptor molecules.

### Investigation of the Effect of Cations Alkyl Chain Length on EPA Extraction

In this study, the capacity values represent the interaction between the chemical structure of ILs and EPA molecules. Further, the COSMO-RS method is used to predict the behaviour of 22 types of anions through the investigation of the effect of increasing cation alkyl chain length base ILs towards EPA in the case of imidazolium, pyridinium, pyrrolidinium, piperidinium, and tetraalkyl ammonium. From the screening, it can be seen that increasing the alkyl chain length in the case of imidazolium cations, had a negative impact on the extraction capacity of the IL (Figure 4 and Supplementary Materials).

This seems true as the capacity could be arranged in the order [EMIM] > [BMIM] > [HMIM]> [OMIM]. However, the IL capacity is remarkably high when combined with the anions, SO_4_^2−^ > Cl^−^ > Br^−^. It is speculated that the interaction between the cation and the anion in these four exceptions is due to the absence of any alky chain in the anion, which makes the interaction (cation-anion) stronger. In addition, the charge density on the cation decreases and the molar volume increases according to the increase in alkyl chain length. The flexible alkyl chain may avoid the ions to pack effectively and cause a decrease in density as the result. It is expected that the increase in alkyl chain length and higher flexibility as the result, may interrupt the movement of IL component and avoid them to pass by each other. Furthermore it may increase the dispersion forces due to the enlarged molecular volume [38]. 

The inorganic ion’s influence seems to be well described by the Hofmeister series. The Hofmeister effect has been studied for decades and in several aspects related to interfacial phenomena such as the behaviour of colloids, the denaturation of proteins, and the structure of microemulsions. The mechanism of the Hofmeister effect is not yet clear, however the notable fact has been proven that the interface within the hydrophilic and hydrophobic environments can be stabilized with presence of ions in the solution and their connections with the charged or polar head groups of amphiphilic molecules [39]. It is also noteworthy that the small anions can easily be replaced by the EPA hydroxyl group.

**Figure 5:**
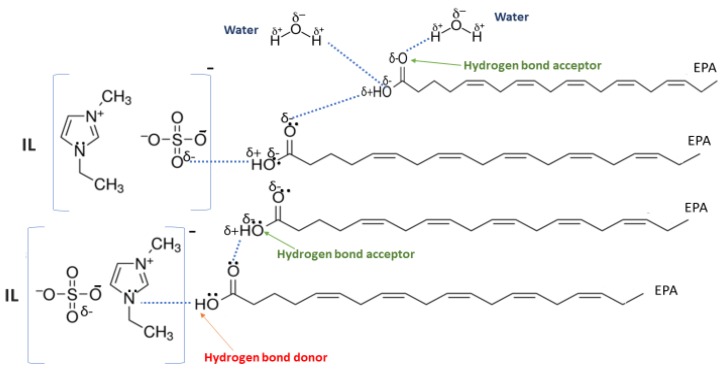
Proposed hydrogen bonding between the IL, [EMIM]^+^SO_4_^2−^, EPA, and water available in the system during extraction.

On the other hand, increasing the alkyl chain length attached to the cation, 3-methyl-pyridinium, contributed to the reduction of the extraction capacity in general, regardless of the anion used. Together, chloride followed by sulfate was the best among other anions tested in the screening. While the capacity value for the pair 1-ethyl-3-methyl-pyridinium and SO_4_ was the highest, a general conclusion cannot be drawn based on the simulation. However, it can be accepted that according to properties of S=O as a good proton acceptor in forming H-bonds with OH from EPA (Figure 5), therefore the hydrogen bond (S=O^…^H) between the cation and anion pair is strong. The regarded fact is even more emphasized while dealing with imidazolium-based IL with SO_4_^2−^ as the anion. The different changing patterns in hydrogen bond strength suggest the possible existence of selective interactions among water, anion, and acidic protons on the imidazolium ring [40].

EPA has two hydrogen bond acceptors and one hydrogen bond donor, and therefore, possibilities to form H-bonding with another EPA molecule is likely to occur during extraction, thus one molecule could attract another molecule, as a chain reaction. The cation, in this case, has no protonated hydrogen, so no hydrogen bond could be from between the alkyl group and the fatty acid. However, it is possible to form H-bond with the nitrogen due to the lone pair. This makes the proposed scheme a likelihood during extraction. 

While poor capacity was observed in case of Br^−^ with [EMPyr], higher values were recorded when paired with [EMPyrro] in the order: SO_4_^2−^ > Cl^−^ > Br^−^ > propanoate. Likewise, [C_n_MPIP] has a similar trend when paired with the anions: SO_4_^2−^, Cl^−^, Br^−^, and propanoate, although the cyclic nature of the cation might slightly reduce the capacity. 

On the other hand, one exception was observed with tetramethylammonium-IL. With the large values of capacity when the anions are paired with SO_4_^2−^, Cl^−^, Br^−^ and propanoate, nitrate [NO_3_^−^] has a different trend from previous cases. The nitrate anion has an electron to donate as well as the lone pair on the nitrogen, which makes it into a potential electron donor (able to bind). Moreover, looking into the tetra-methyl ammonium cation, it has positively charged nitrogen as all electrons are occupied with methyl groups. This may explain the low capacity of the pair (tetra-methyl ammonium nitrate) in comparison to Cl^−^, Br^−^ which possess higher electronegativity. 

It may be predicted that in case larger alkyl groups, the anion can be at a more stable position, which could hinder its capacity for fatty acids extraction. Another suggestion is that ILs with hydrophobic anions (such as NTF_2_) and long alkyl chain are a better choice for the extraction of non-polar substances, such as EPA, as the long carbon chain reduced the polarity of the FFA. On the other hand, ILs such as [EMIM]Cl are highly hydrophilic. 

In this situation, the correlation between n-3 PUFA extraction and ionic liquids structures (aromatic or delocalized cation) for more enhanced PUFA extraction was suggested by Cheong et al. (2011) [41]. The hydrophilic or hydrophobic characteristics of ILs is significantly dependent on the structures of cations and anions, however the water miscibility of ILs are highly associated with the anion portion. The IL based on [PF_6_]^−^ and [Tf_2_N]^−^ are typically water-immiscible and are therefore desirable for forming biphasic systems. The regarded ILs are widely used for the target of extraction [42]. Li et al., (2009) was the first to report a study on the recovery of n-3 PUFA methyl esters from fish oil in which the ILs were combined with silver salts, and as the result, the hydrophobic ILs with large anions of low lattice energy were selected as the most desirable output [43,44]. Viscosity plays a crucial role, in the extraction of which, lower viscosity extensively improves the process. Moreover from the results, it was observed that the extraction capability of pyrrolidinium and tetra ammonium-based ILs were considerably lower than imidazolium and pyridinium-based ILs as it was below 20% for n-3 PUFAs and lower than 6% for n-3 PUFAEEs.

It can be noticed from these findings that the enrichment efficiency of n-3 PUFAs are strongly correlated to the aromatic structure of ILs. The correlation can be explained as the IL with aromatic structure is able to form temporary weak π-π interactions with polyunsaturated bonds in n-3 PUFA and enriches the component in the IL phase. It is found that ILs with cations containing aromatic rings, namely, the N,N′-dialkylimidazolium ([BMIM]PF_6_, [OMIM]PF_6_, [BMIM]BF_4_, [OMIM]BF_4_, and [BMIM]OTF) and N-alkylpyridinium ([BuMePyr]DCN, [BuMePyr]OTF, and [MeOcPyr]BF_4_) types have higher selectivity and extraction capability for n-3 PUFA and n-3 PUFAEE, as compared to those without aromatic structure. It can be concluded that the selectivity increases and hence the extraction capability improves with alkyl chain length from C_4_ to C_8._ However, it is not yet accurate to select the IL with higher selectivity merely by looking through cations. For example, in a study by Reference [41], same cation was used with different anions in screening ILs and it was observed that smaller anions have better extraction capability for PUFA. Fluoride (F^−^) has the highest electronegative value and has the smallest anion size among the other anions in the comparison, [F^−^ = Ne = 1s^2^2s^2^2p^6^], which might be one of the reasons of its extraction capability, besides the stable electron configuration (the corresponding noble gas), although the COMSO-RS analysis demonstrated the opposite. 

Table 1 summarize Figure 4a–e for the ILs with high potential of EPA extraction in terms of infinite dilution activity coefficient and capacity values. In the following table, there is shortlist of the ILs with high capacity and lower activity coefficient at infinite dilution. Activity coefficient at infinite dilution is a term firstly introduced and discussed by Reference [45]. In this study, the activity coefficient is a term to account for the effective concentration of a solute in a solution of which its chemical potential can be expressed by the activity of the substances present in the solution. When the concentration of the solute tends to zero, the terms are hence called the activity coefficient at infinite dilution [46,47]. Activity coefficient at infinite dilution provides a deep vision on the degree of non-ideal behaviour in ILs–EPA compounds mixtures. Low activity coefficient values show high solubility of the ILs with weak solute-ILs interaction and vice versa, as described by References [48,49]. 

According to the listed ILs in Table 1, mono-cations were the selected cations since the anion (SO_4_^2−^) with the charge of (−2) is outstanding and hence the overall charge for the IL molecule will be (−1). The presence of free charge is expected to increase the polarity and therefore increase the solvent extractive particularly.

The final conclusion about the rate of mass transfer, the solubility of all components of the model solution, the distribution coefficients, and selectivity must be determined in order to decide the suitable IL for extraction [50].

## 3. Methodology

### 3.1. Computational Details of COSMO-RS

The molecules geometries were optimized by turbo mole 6.3 program package (TURBOMOLE GmbH (ltd), Univerisity of Karlsruhe Karlsruhe, Germany) that follows the density functional theory (DFT), to generate COSMO files using Becke and Perdew (BP) functional B88-86 with a triple-zeta valence with polarization (TZVP), and the resolution of identity standard (RI) approximation as a basis set [51]. 

COSMO-RS is a theory with the purpose of quantum theory, surface interactions, dielectric continuum models, and statistical thermodynamic properties that has been described for the first time in a work by Reference [23]. At first, in 1998 the application of COSMO-RS was limited by calculation of the activity coefficient in infinite dilution for vapour-liquid equilibria of binary mixtures. Then, the usage of COSMO-RS go further to calculate all kinds of phase equilibrium such as vapour-liquid, liquid-liquid, and solid-liquid predictions [52,53].

**Figure 6:**
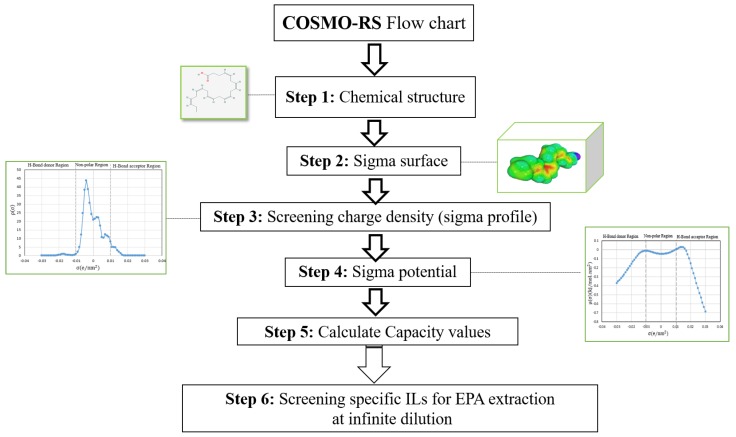
COSMO-RS calculation steps.

In this study, the COSMO-RS approach has been used to screen 352 ILs (22 anions-16 cations combination) and to calculate their related capacity values. Selected ILs (with higher capacity values) are intended to be applied as solvents in EPA extraction. Figure 6 illustrates the following steps for screening, and therefore, the selection of an appropriate IL to be used in the extraction of the total EPA compound.

### 3.2. Data Collection and Screening Process

The chemical structure of EPA which is illustrated in Table 2 is first optimized by TZVP according to thermodynamic properties and inserted to the data bank of COSMO-RS. Next, cations were selected from imidazolium, pyridinium, pyrrolidinium, piperidinium, and tetra-methyl ammonium based-ILs and hydrophilic and hydrophobic anions. The screened anions and cations based ILs were referred from the lipid extraction literature as a basis, as shown in Table 3 and Table 4. Then, the software estimates the sigma profile and sigma potential of EPA and calculates the related capacity values for each IL toward EPA compound.

### 3.3. COSMO-RS Assumptions

Solvent extraction process is a robust and economical methodology to be applied for extraction of EPA. There are three assumptions that were made during the screening process including: (a) The liquid state is incompressible, (b) all parts of the molecular surfaces can be in contact with each other, and (c) only pairwise interactions of molecular surface patches are allowed. 

With respect to the stated assumptions, screening of charge density and capacity value at infinite dilution were performed by COSMO-RS. The significance of capacity value at infinite dilution is where it defined whether the targeted solvent has adequate strength in extraction. The screening charge density is defined as sigma profile of mixture $\left(p_{s}\left(\sigma \right)\right)$ and it can be calculated by Equations (1) and (2) [23,54,55]:

(1) $$p_{s}\left(\sigma \right)=\sum_{i=s}X_{i}\;p^{X_{i}}\left(\sigma \right)$$

(2) $$p^{X_{i}}\left(\sigma \right)=\frac{n_{i}\left(\sigma \right)}{n_{i}}=\frac{A_{i}\left(\sigma \right)}{A_{i}}$$

In Equation (1), $X_{i}$ is the mole fraction of compound *X*, and $p^{X_{i}}\left(\sigma \right)$ is the sigma profile of compound *X*. Additionally, in Equation (2), $n_{i}(\sigma )$ is the number of the segment with the surface charge density σ, $A_{i}(\sigma )$) is the total surface area of all segment with particular charge density σ. 

The sigma potential ($\mu_{s}\left(\sigma \right))$ rely on composition, temperature (*T*), sigma profile $\left(P_{s}\right),\;$and electrostatic interaction energies ($E_{misfit})$, which is determined by Equation (3):

(3) $$\mu_{s}\left(\sigma \right)=-\frac{RT}{a_{eff}}ln\left[\int P_{s}\left(\sigma^{\prime }\right)exp\left(\frac{a_{eff}}{RT}\left(\mu_{s}\left(\sigma^{\prime }\right)-E_{misfit}\left(\sigma ,\sigma^{\prime }\right)-E_{HB}\left(\sigma ,\sigma^{\prime }\right)\right)\right)d\sigma^{\prime }\right]$$

Equations (4) represent the electrostatic interaction energies [56].

(4) $$E_{misfit}\left(\sigma ,\sigma^{\prime }\right)=a_{eff}\frac{\alpha^{\prime }}{2}{\left(\sigma +\sigma^{\prime }\right)}^{2}$$

where R is ideal gas constant, T is also absolute temperature, $a_{eff}$ is the effective area of contact, $E_{HB}$ is hydrogen bonding energy, $E_{misfit}\;$is electrostatic interaction energy of two segments per unit area, *α*′ is an interaction parameter, *σ* and *σ*′ are two different screening charge densities for solute molecules that are in contact with each other, and $E_{misfit}\;$is the electrostatic interaction energy of two segments per unit area. 

The solvent capacity accounts for the quantity of solute that has been removed from the mixture by the extracting solvent [57]. The solvent capacity at infinite dilution, is numerically the maximum amount of solute (EPA) that can be dissolved in the solvent (IL). The capacity values of ILs for the extraction of EPA calculated by Equation (5).

(5) $$C^{\infty }={\left(\frac{1}{\gamma^{\infty }}\right)}^{Ionic\;liquid\;phase}$$

where, $\gamma^{\infty }$ is the activity coefficient of solvent at infinite dilution. The value of capacity indicates the amount of solvent that is used during the separation process. The higher solvent capacity results in a lower amount of solvent used. Therefore, the solvent with highest capacity value tends to be more desirable to choose as a separating agent.

## 4. Conclusions

Overall, the COSMO-RS method was used to screen the suitable ILs for microalgae lipid extraction based on the findings from this study. The highest capacity for EPA extraction among 352 cation/anion combinations was belong to [TMAm][SO_4_] followed by [BMPIP][SO_4_] < [EMPyr][SO_4_] < [EMIM][SO_4_]<[EMPyrro][SO_4_] < [TMAm][SO_4_] by the order of low to high. It is expected to apply this combination ([TMAm][SO_4_]) to produce a high yield of EPA from microalgae. It is expected to have a better extraction with the small anions that possess high charge density compared to larger ones. Moreover, shorter alkyl chain cations are preferred when using imidazolium-based IL, which agrees with experimental data. Overall, we may conclude that other factors should be addressed to select the IL for extraction such as the rate of mass transfer, the solubility, selectivity, and viscosity. Indeed, screening for the most suitable ILs for extraction reduces the time required and therefore would be more efficient in terms of resources. 

## Figures and Tables

**Table 1:** Summarize the selective ILs for EPA extraction.

#	Shortlisted ILs	Infinite Dilution Activity Coefficient at 298.15 K	Infinite Dilution Capacity at 298.15 K
1	[TMAm]SO_4_	1.00 × 10^−11^	9.97 × 10^10^
2	[TMAm]Cl	9.52 × 10^−10^	1.05 × 10^9^
3	[TMAm]Br	3.61 × 10^−8^	27677690
4	[EMPyrro]SO_4_	4.40 × 10^−7^	2273253
5	[EMPyrro]Cl	1.68 × 10^−5^	59473.56
6	[EMIM]SO_4_	1.87 × 10^−5^	53360.83
7	[MPPIP]SO_4_	4.17 × 10^−5^	23995.28
8	[EmPyr]SO_4_	7.10 × 10^−5^	14084.39
9	[BMPyrro]SO_4_	9.03 × 10^−5^	11073.19
10	[BPPIP]SO_4_	2.13 × 10^−4^	4689.32
11	[EMPyrro]Br	2.41 × 10^−4^	4147.65
12	[EMIM]Cl	6.61 × 10^−4^	1513.22
13	[TMAm]propanoate	6.97 × 10^−4^	1434.47
14	[TMAm]NO_3_	8.39 × 10^−4^	1191.69
15	[EMPyrro]propanoate	8.67 × 10^−4^	1153.16
16	[HMPyrro]SO_4_	9.62 × 10^−4^	1039.60
17	[MPPIP]Cl	1.01 × 10^−3^	991.30
18	[BMIM]SO_4_	1.28 × 10^−3^	781.77
19	[HPPIP]SO_4_	1.28 × 10^−3^	781.45
20	[BmPyr]SO_4_	1.37 × 10^−3^	727.47
21	[EmPyr]Cl	1.74 × 10^−3^	575.71

**Table 2:** Structure of EPA.

Shorthand Sign	Synthetic Name	Trivial Name	Formula	Chemical Structure
20:5	Eicosapentanoic acid	EPA	C_20_H_30_O_2_	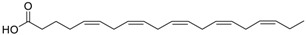

**Table 3:** The screened anions used in this study.

No.	Anions/Abbreviations	Chemical Structure
1	Chloride [Cl]	Cl^−^
2	Bromide [Br]	Br^−^
3	Tetrafluoroborate [BF_4_]	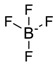
4	Hexafluorophosphate [PF_6_]	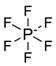
5	Nitrate [NO_3_]	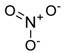
6	Dicyanamide [DCN]	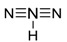
7	Diethylphosphate [C_4_H_10_O_4_P]	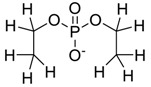
8	Tetrachloro aluminate [AlCl_4_]	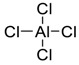
9	Methyl sulfate [CH_4_O_4_S]	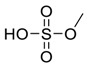
10	Thiocyanate [SCN]	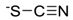
11	Methane sulfonate [C_2_H_6_O_3_S]	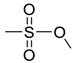
12	Ethyl sulfate [C_2_H_6_O_4_S]	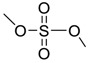
13	Benzoate [C_7_H_5_O_2_]	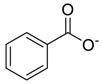
14	Sulfate [SO_4_]	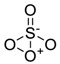
15	Hydrogen sulphate [HSO_4_]	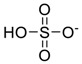
16	Dimethyl phosphate [C_2_H_7_O_4_P]	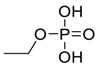
17	Propanoate [C_3_H_5_O_2_]	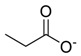
18	Toluene-4-Sulfonate [C_7_H_7_O_3_S]	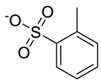
19	Tri fluoro methane-Sulfonate [CF_3_SO_3_]	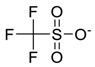
20	Bis(Trifluoromethyl)Imide [NHC_2_F_6_]	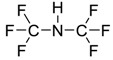
21	Trifluoroacetate [CF_3_CO_2_]	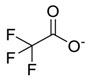
22	Bis(trifluoromethylsulfonyl) imide [TF_2_N]	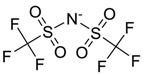

**Table 4:** The screened cations used in this study.

No.	Cations/Abbreviations	Chemical Structures
1	1-ethyl-3-methyl imidazolium [EMIM]	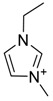
2	1-butyl-3-methyl imidazolium [BMIM]	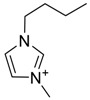
3	1-hexyl-3-methyl imidazolium [HMIM]	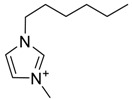
4	1-octyl-3-methyl imidazolium [OMIM]	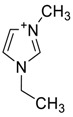
5	1-ethyl-3-methyl pyridinium [EMPyr]	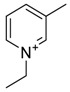
6	1-butyl-3-methyl pyridinium [BMPyr]	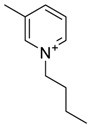
7	1-hexyl-3-methyl pyridinium [HMPyr]	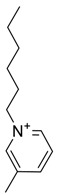
8	1-octhyl-3-methyl pyridinium [OMPyr]	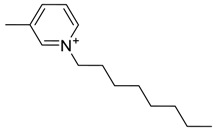
9	1-butyl-1-methyl pyrrolidinium [BMPyrro]	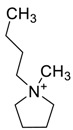
10	1-ethyl-1-methyl pyrrolidinium [EMPyrro]	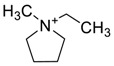
11	1-hexyl-1-methyl pyrrolidinium [HMPyrro]	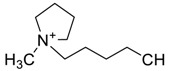
12	1-methyl-1-octyl pyrrolidinium [MOPyrro]	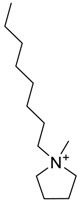
13	1-butyl-1-methyl piperidinium [BMPIP]	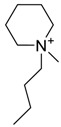
14	1-hexyl-1-methyl piperidinium [HMPIP]	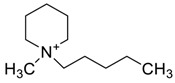
15	1-methyl-1-propyl piperidinium [MPPIP]	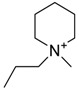
16	Tetra-methyl ammonium [TMAm]	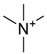

## Supplementary Materials

The following are available online at http://www.mdpi.com/1420-3049/24/4/713/s1.
