# Supplementary material for: Screening of Suitable Ionic Liquids as Green Solvents for Extraction of Eicosapentaenoic Acid (EPA) from Microalgae Biomass Using COSMO-RS Model
Source: Molecules. 2019 Feb 16;24(4):713. doi: 10.3390/molecules24040713 (PMC6412376; doi:10.3390/molecules24040713)
Supplement: Supplementary file 1 [file molecules-24-00713-s001.pdf]

Supplementary Materials

Full report of screening capacity values of 352 ILs through COSMO-RS.

Table S1. Summary of Infinite dilution capacity values for the screened imidazolium-based ILs

	Infinite dilution capacity values at 298.15			
**Anions/cations**	**[EMIM]**	**[BMIM]**	**[HMIM]**	**[OMIM]**
Cl	1513.223	36.69524	9.283301	5.592392
Br	97.16176	4.144935	1.58632	1.284895
BF_4	0.012887	0.03788	0.108393	0.250704
PF_6	0.002406	0.031112	0.127148	0.308284
NO_3	1.059998	0.487667	0.609569	0.93394
DCN	0.050434	0.163087	0.412812	0.836898
Diethylphosphate	5.903097	6.810307	8.119651	9.593417
Tetrachloroaluminate	0.065825	0.269331	0.590124	0.946038
Methylsulfate	0.277864	0.304479	0.446189	0.682384
Thiocyanate	0.11663	0.166223	0.353411	0.712117
Methanesulfonate	6.556698	2.302389	1.885866	2.077005
Ethylsulfate	0.291224	0.412874	0.61898	0.908798
Benzoate	9.085931	12.64911	17.44019	22.76165
SO_4	53360.83	781.7713	140.0588	65.86797
HSO_4	0.045978	0.042309	0.077279	0.151674
Dimethylphosphate	7.217706	5.792229	6.118012	7.046332
Propanoate	80.34559	46.44307	42.20916	43.19424
Toluene-4-sulfonate	0.624033	1.002731	1.473628	2.042342
Trifluoromethane-sulfonate	0.038224	0.153206	0.367952	0.672971
Bis(trifluoromethyl)imide	0.049493	0.400492	1.21611	2.372792
Trifluoroacetate	0.324304	0.87971	1.783053	2.954198
TF_2N	0.079627	0.226989	0.429814	0.655981

Table S2. Summary of Infinite dilution capacity values for the screened pyridinium-based ILs

	Infinite dilution capacity values at 298.15			
**Anions/cations**	**[EMPyr]**	**[BMPyr]**	**[HMPyr]**	**[OMPyr]**
Cl	575.7118	44.88336	15.35079	10.40293
Br	53.82011	6.623389	3.121854	2.617886
BF_4	0.057588	0.136887	0.293537	0.523194
PF_6	0.013208	0.080488	0.234558	0.444541
NO_3	2.045367	1.400245	1.615876	2.146239
DCN	0.197205	0.492563	0.972784	1.642332
Diethylphosphate	12.14281	14.13575	15.85107	17.79019
Tetrachloroaluminate	0.098206	0.307213	0.599414	0.862429
Methylsulfate	0.702063	0.811864	1.060221	1.415577
Thiocyanate	0.377365	0.551876	0.941847	1.544387
Methanesulfonate	9.995822	5.039854	4.170881	4.35732
Ethylsulfate	0.75054	1.025384	1.369529	1.783339
Benzoate	22.04481	29.42301	36.45969	44.62112
SO_4	14084.39	727.4697	189.7338	109.352

HSO_4	0.117249	0.134582	0.214705	0.351656
Dimethylphosphate	14.126	12.52977	12.62371	13.77723
Propanoate	137.2628	93.93727	82.84796	83.72329
Toluene-4-sulfonate	1.441692	2.174083	2.900135	3.69004
Trifluoromethane-sulfonate	0.135281	0.385736	0.74955	1.163622
Bis(trifluoromethyl)imide	0.173334	0.83556	2.013606	3.328884
Trifluoroacetate	1.108078	2.331256	3.87803	5.575752
TF_2N	0.145837	0.332897	0.559662	0.771875

Table S3. Summary of Infinite dilution capacity values for the screened pyrrolodinium-based ILs

	Infinite dilution capacity values at 298.15			
**Anions/cations**	**[EMPyrro]**	**[BMPyrro]**	**[HMPyrro]**	**[MOPyrro]**
Cl	59473.56	454.8407	58.20884	23.69128
Br	4147.65	55.54687	10.58457	5.691747
BF_4	0.195049	0.1502383	0.228056	0.3830986
PF_6	0.006198	0.0380489	0.110454	0.226504
NO_3	36.1974	4.399836	2.726823	2.86096
DCN	1.23805	1.337681	1.924791	2.845495
Diethylphosphate	30.29817	26.20518	25.32994	26.4794
Tetrachloroaluminate	0.031465	0.1343895	0.279486	0.4291654
Methylsulfate	3.316457	1.567978	1.423954	1.651555
Thiocyanate	3.76933	1.493462	1.643926	2.310415
Methanesulfonate	110.1086	16.39091	8.148609	6.68387
Ethylsulfate	2.11473	1.66517	1.738786	2.059302
Benzoate	110.8388	91.74538	91.99159	100.6277
SO_4	2273253	11073.19	1039.601	337.9954
HSO_4	1.119069	0.2993707	0.28333	0.387212
Dimethylphosphate	67.29441	30.7284	23.05741	21.79057
Propanoate	1153.155	346.1794	220.7801	189.2232
Toluene-4-sulfonate	3.591849	3.921982	4.402388	5.153351
Trifluoromethane-sulfonate	0.165994	0.3922865	0.690802	1.06722
Bis(trifluoromethyl)imide	0.076541	0.5131368	1.338162	2.412847
Trifluoroacetate	2.776517	3.616502	5.062686	7.000539
TF_2N	0.094677	0.24152	0.40589	0.5710418

Table S4. Summary of Infinite dilution capacity values for the screened piperidinium-based ILs

	Infinite dilution capacity values at 298.15		
**Anions/cations**	**[MPPIP]**	**[BMPIP]**	**[HMPIP]**
Cl	991.3	230.7921	49.66533
Br	119.2363	34.45304	10.2776
BF_4	0.2231786	0.2382243	0.3490002
PF_6	0.03959182	0.07154971	0.1672137
NO_3	7.877709	4.729489	3.505549
DCN	1.763459	1.933201	2.655246
Diethylphosphate	33.32664	31.14698	30.4196

Tetrachloroaluminate	0.1166237	0.1877521	0.3386179
Methylsulfate	2.345239	1.940794	1.863241
Thiocyanate	2.340342	2.026676	2.319745
Methanesulfonate	27.72147	15.71487	9.391614
Ethylsulfate	2.271152	2.132355	2.251591
Benzoate	116.3144	108.5453	109.4828
SO_4	23995.28	4689.317	781.4509
HSO_4	0.4806515	0.375787	0.3945053
Dimethylphosphate	43.86634	33.89567	27.27027
Propanoate	497.5008	341.2575	241.4898
Toluene-4-sulfonate	4.840641	4.932457	5.46939
Trifluoromethane-sulfonate	0.4465639	0.5926313	0.9409241
Bis(trifluoromethyl)imide	0.476145	0.8547444	1.830789
Trifluoroacetate	4.525903	5.049439	6.680796
TF_2N	0.2323936	0.3170129	0.4854676

Table S5. Summary of Infinite dilution capacity values for the screened tetra methyl ammonium-based ILs

	**Infinite dilution capacity values at 298.15**
**Anions/cations**	**[TMAm]**
Cl	1.05E+09
Br	2.77E+07
BF_4	5.42E-02
PF_6	4.10E-06
NO_3	1.19E+03
DCN	7.90E-02
Diethylphosphate	4.66E+00
Tetrachloroaluminate	9.67E-03
Methylsulfate	1.33E+00
Thiocyanate	8.00E+00
Methanesulfonate	6.19E+02
Ethylsulfate	2.69E-01
Benzoate	1.33E+01
SO_4	9.97E+10
HSO_4	6.31E+00
Dimethylphosphate	2.71E+01
Propanoate	1.43E+03
Toluene-4-sulfonate	3.36E-01
Trifluoromethane-sulfonate	2.14E-03
Bis(trifluoromethyl)imide	5.27E-04
Trifluoroacetate	9.79E-02
TF_2N	1.02E-02
